# Application of Epigallocatechin-3-gallate (EGCG) Modified 1-Ethyl-3-(3-dimethylaminopropylcarbodiimide hydrochloride/N-hydroxy-succinimide (EDC/NHS) Cross-Linked Collagen Membrane to Promote Macrophage Adhesion

**DOI:** 10.3390/ma14164660

**Published:** 2021-08-18

**Authors:** Shengan Rung, Xiwen Zhao, Chenyu Chu, Renli Yang, Yili Qu, Yi Man

**Affiliations:** 1 State Key Laboratory of Oral Diseases, West China Hospital of Stomatology, Sichuan University, Chengdu 610041, China; 2017151642144@stu.scu.edu.cn (S.R.); zhaoxiwen@stu.scu.edu.cn (X.Z.); zhuchenyou@stu.scu.edu.cn (C.C.); renliyang@stu.scu.edu.cn (R.Y.); 2 Department of Oral Implantology, West China Hospital of Stomatology, Sichuan University, Chengdu 610041, China

**Keywords:** collagen membrane, EGCG, adhesion, macrophage, implant dentistry

## Abstract

The chemically cross-linking 1-ethyl-3-(3-dimethylaminopropylcarbodiimide hydrochloride/N-hydroxy-succinimide (EDC/NHS) collagen membrane endows such natural polymers with promising mechanical properties. Nevertheless, it is inadequate to advance the modulation of foreign body response (FBR) after implantation or guidance of tissue regeneration. In previous research, macrophages have a strong regulatory effect on regeneration, and such enhanced membranes underwent the modification with Epigallocatechin-3-gallate (EGCG) could adjust the recruitment and phenotypes of macrophages. Accordingly, we develop EGCG-EDC/NHS membranes, prepared with physical immersion, while focusing on the surface morphology through SEM, the biological activity of collagen was determined by FTIR, the activity and adhesion of cell culture in vitro, angiogenesis and monocyte/macrophage recruitment after subcutaneous implantation in vivo, are characterized. It could be concluded that it is hopeful EGCG-EDC/NHS collagen membrane can be used in implant dentistry for it not only retains the advantages of the collagen membrane itself, but also improves cell viability, adhesion, vascularization, and immunoregulation tendency.

## 1. Introduction

Guided tissue regeneration (GTR) and guided bone regeneration (GBR) surgery take advantage of barrier membrane to compartmentalize soft and hard tissue [1]. Currently, as the key organic component of extracellular matrix (ECM), type I and III collagen are the main component of the barrier membrane with the survival rate close to 100% [2]. Without the addition of crosslinking agents, the membrane may not be able to provide sufficient support as well as the ability of regulating an immune microenvironment [3]. The transformation of macrophages M1 phenotype (pro-inflammatory) and M2 phenotype (pro-repair) in an immune microenvironment has become an important target for promoting regeneration and repair, which can regulate the immune microenvironment to a certain extent, and promote the transformation of tissues in the direction of repair and regeneration. Among the cross-linking components of collagen membranes used in regeneration, 1-ethyl-3-(3-dimethylaminopropyl) carbodiimide hydrochloride (EDC) and N-hydroxy-succinimide (NHS), are covalently bonded, which has good biocompatibility and non-toxicity, with mechanical properties improved, structure maintained in vivo, and cell proliferation benefited [4,5,6,7]. However, introduction to the membrane elicits the material-dependent inflammatory response called foreign body response (FBR), a significant balanced reaction affecting regeneration outcome [5,6]. As a foreign biological material implanted, the barrier membrane would inevitably trigger the host membrane immune response after implantation, which requires the activation of phagocytes and the Th2 cells releasing IL-3 and introducing the formation of foreign body giant cells (FBGCs), leading to the degradation of collagen membranes and enhancement of phagocytic capability [7]. Therefore, it is important to develop a membrane that utilizes immune regulation to promote the reduction of local foreign body reactions. Especially in periodontitis, a chronic and degenerative inflammatory disease which affects approximately 10% of the world’s population, bacteriophages may be more involved in periodontal disease than previously thought, because they are known to have significant changes in the bacterial composition of moderate to severe periodontitis [8,9]. The inflammatory microenvironment makes it harder to conduct tissue engineering with biomaterials, thus elevates the importance of immune modulation to rebuild balance [5]. Macrophages, the dominant cells in FBR, whose transformation from pro-inflammatory towards pro-regenerative phenotype are vital in regeneration and wound healing after materials implantation [10]. If the material has the ability to adjust such interaction by providing suitable immune microenvironment, not only the pro-regenerative macrophage, but also the guidance of other cells that prompt to repair will accumulate in sequence. The single-cell sequencing has also revealed the transformation of macrophage on account of tissue regeneration and wound healing [11], which would promote the expression of T helper cells and reduce inflammation [11,12].

**Figure 1:**
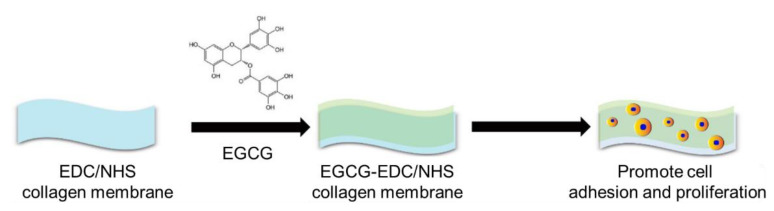
Schematic graph of loading 0.064% EGCG to EDC/NHS collagen membrane.

As one of the main polyphenols extracted from tea, epigallocatechin-3-gallate (EGCG) could serve as a crosslink agent for scaffolds, showing bioactive effects in various aspects, especially in dental restoration [13]. Compared with pure collagen membrane, its anti-inflammatory [14], anti-fibrosis [15], pro-osteogenic [16], vascularization [17], and macrophage phenotypes regulatory [11] effects are all a prompt to rescue the prolonged inflammation and help the establishment of an ideal microenvironment to provide a promising signal in regeneration [18]. The literature has shown that macrophages switch from M1 to M2 to promote osteogenesis [19]. Considering its promising traits, EGCG was attached to the commercial EDC/NHS collagen, and the modification of EGCG was adjusted at 0.064% *w*/*v*, where it is reported to possess appropriate mechanical properties, cell viability promotion and anti-inflammatory effects in previous report [20]. In order to render the developed EDC/NHS collagen membrane modified by EGCG meets the need of GBR/GTR, its physical, chemical, and biological properties were characterized by means of an investigation on surface morphology, FTIR spectra, DSC statistics, in vitro cell viability and integrin expression, and in vivo vascularization and monocyte/macrophage recruitment. The aim of this study is to fabricate 0.064% EGCG EDC/NHS collagen membranes (Figure 1) with the improvement of cell viability and adhesion, as well as revascularization. 

## 2. Materials and Methods

### 2.1. Materials

Commercial EDC/NHS collagen membranes (Dentium, Gyeonggi-do, Korea) of a size of 10 mm × 20 mm were purchased, whereas EGCG was bought in powder (Jiang Xi Lv Kang Natural Products, China). The EGCG modified EDC/NHS collagen membrane was fabricated as follows, which refers to our pervious study [20]: each EDC/NHS collagen membrane was immersed in 0.064% *w/v* EGCG solution for 1 h at room temperature. After this procedure, membranes were rinsed in deionized water three times and freeze dried overnight. The pure EDC/NHS collagen membranes were also processed under the same protocol except for immersion.

### 2.2. Surface Morphology

Accelerating voltage of 25 kV was adopted to characterize surface morphology via scanning electron microscope (SEM, S-800, HITACHI, Tokyo, Japan). The discs were sprayed with Au/Pt in an ion sputter (E1010, HITACHI, Tokyo, Japan) to gain adequate electrical conductivity.

### 2.3. Mechanical and Chemical Properties

a. Mechanical properties measurement

The ultimate elongation (UE), ultimate stress (US), and Young’s modulus (YM) of the membranes were obtained from an electronic universal test machine (SHIMADZU, AG-IC 50KN, Kyoto, Japan) with the average of five specimens (10 mm × 20 mm) for each kind of membrane. Specimens were strained at room temperature with a speed of 15 mm/min. When operating differential scanning calorimeter (DSC) measurement, samples with a size of 10 mm × 20 mm (~6.71 mg) were encapsulated using aluminum pan, then heated to 500 °C at a rate of 10 °C/min in a nitrogen atmosphere. The Netzsch Proteus analysis software was used to attain the thermo-grams, as the typical peak of it was interpreted as thermal denaturation.

b. Chemical properties measurement

A Fourier transform infrared spectroscopy (FTIR) spectrophotometer (Spectrum One, PerkinElmer, Inc., Waltham, MA, USA) was employed at 32 scans on average to measure the FTIR spectra of the samples, the spectra of which were obtained at room temperature within 400–4000 cm^−1^.

c. Release profile of EGCG-EDC/NHS-Col in vitro

EGCG-EDC/NHS-Col was soaked in PBS in 48-well plate at 37 °C, 5% CO_2_, and scanned by ultraviolet-visible spectrophotometry to obtain the release profile. An OD value of 270 nm was used to detect EGCG in solution. At 0 h, the instrument was calibrated to zero, then, measured the OD value of the media at 2, 12, 48, 72, 120, 168, and 216 h by removing media (200 μL) and replenishing with fresh buffer (200 μL) each time.

### 2.4. Cell Viability

The examination employed murine macrophage cell line, Raw 264.7 (American Type Culture Collection, Rockville, MD, USA). Staining of Cell Counting Kit-8 (CCK-8, Dojindo Laboratories, Kumamoto, Japan) and Calcein AM/Hoechst were both conducted, but for different time periods. For CCK-8, membranes were processed into 10 mm × 10 mm samples on the clean bench, then placed in 48-well plates and seeded with Raw 264.7 cell at a density of 10^4^/well. Each well was be supplemented with 10% CCK-8 solution. Cells were then co-cultured for 10, 20, 40, 60, 120, 240, 360 min, and 1 day, respectively, with RPMI (Gibco, Thermo, Waltham, MA, USA) mixed with 10% FBS (Gibco, Thermo, Waltham, MA, USA). Then plates would be incubated at 37 °C for another 3.5 h. After incubation, the cell viability was determined through a micro-plate reader (Multiskan, Thermo, Waltham, MA, USA) via measuring the OD value at 450 nm. As for staining of Calcein AM/Hoechst, cells were cultured for 5 days on the dishes then incubated with 1 μM Calcein AM (diluted from a 1 mM stock solution of CAM in dimethyl sulfoxide, Dojindo Laboratories, Kumamoto, Japan) and 100 μL RPMI for 30 min in the incubator. The similar procedure cultured cells for 5 days and stained with 0.5 μM PI (diluted from a 0.5 mM stock solution of PI in dimethyl sulfoxide, Dojindo Laboratories, Kumamoto, Japan) incubating and 100 μL RPMI for 2 min in the incubator. Then, the dishes were rinsed with 1×PBS three times and stained with 100 μL Hoechst 33,258 working fluid (diluted from a 1 mL stock solution in distilled water, KeyGen, Bejing, China) for 10 min in the incubator. At last, the dish was washed three times with 1×PBS and analyzed by an Inverted Ti-E microscope (Nikon, Tokyo, Japan). The number of the cells were counted using image analysis software ImageJ 1.50i (2016, National Institute of Health, Bethesda, MD, USA). Cell counts were measured by *t*-test. 

### 2.5. Cell Adhesion

SEM images were taken to preliminary estimate cell adhesion after culturing on EDC/NHS-Col and 0.064% EGCG-EDC/NHS-Col for 10, 20, 40, and 480 min, with processed as mentioned in Section 2.2. The expressions of integrin beta 1, 2, and 3 in Raw 264.7 cells after culturing on blank wells, EDC/NHS-Col, and 0.064% EGCG-EDC/NHS-Col for 10 min were further evaluated by RNA isolation, cDNA synthesis, and RT-qPCR. Cells cultured on different surfaces were collected after incubating with 0.05% trypsin-EDTA (Gibco, Thermo, USA) for 5 min and homogenized in 1-mL Trizol Reagent (Tianjian, Beijing, China). The mRNA Selective PCR kit (TaKaRa Bio-Clontech) was used to reverse-transcribe the whole RNA. Mouse integrin beta 1, 2, and 3 cDNA were amplified through real-time PCR by using the SYBR Green PCR kit (Thermo Scientific, Braunschweig, Germany). The primer sequences, which were used for the real-time PCR, were listed in Table 1.

### 2.6. Surgical Procedures

The protocol of this present experiment was approved by Institution Review Board of West China Hospital of Stomatology (No.WCHSIRB-D-2017-097). Male C57BL/6, 6~7 weeks of age, were adaptively fed for 3 days after purchasing. After anaesthetization, the surgical area of the back was shaved and prepared aseptically. Three parallel sagittal incisions were made in the dorsal skin and membrane implantation was prepared by subcutaneous pockets. The two kinds of membranes were prepared beforehand into 3 mm × 3 mm size in the clean bench. EDC/NHS collagen membranes and EGCG-EDC/NHS collagen membranes were respectively implanted into these pockets. The control group underwent a sham procedure. Afterwards, all the incisions were sutured. These mice were professionally kept in the strict experimental animal room and were fed with the standard laboratory diet. After recovering for 0 (12 h), 1, and 3 days, they were killed by cervical dislocation. The membranes and the skins covering the materials were harvested together (equal areas around the suture were harvested in the control groups) and immediately fixed with 4% paraformaldehyde (Solarbio, Beijing, China) for at least 24 h. After confirming the surgical site, hair was removed, and a round full-thickness skin defect with a diameter of 6 mm was prepared on the back of the murine. Appropriate blunt dissection is performed subcutaneously around the defect to ensure smooth implantation of the membrane underneath it. After that, the silicone splints and the defect circumference were fixed to ensure that the wound had no tension, and the healing was process under the condition of no stress [10]. Samples were then collected at 12 and 24 h for qPCR detection.

### 2.7. HE Staining

The fixed samples were embedding in paraffin and then sliced into 3-μm thick sections for the following immunofluorescence assay and HE staining. While staining with HE dyes solution, these sections were incubated for 4 h at 65 ℃ to deparaffinize and processed as follow:

(a) Dehydration with ethanol;
(b) Stain for 5 min using hematoxylin and differentiate for 2 s in 1% hydrochloric acid alcohol;
(c) Incubate for 2 min in 0.2% ammonia water and stain for 1 min using eosin;
(d) Gradually dehydrate using 95% ethanol, then clear and mount the sections with neutral resin. These sections were observed with light microscopy (Olympus, Tokyo, Japan) and scanned by the Digital Slide Scanning System (PRECICE, Beijing, China).

### 2.8. Promotion of Revascularization

Three days after implantation, tissue was harvested and prepared as sections. Then, sections would be fixed with 2% paraformaldehyde PBS for 24 h (pH = 7.4) and further washed by PBS 3 times, which was followed by soaking in 1% bovine serum albumin (BSA) PBS that contained 1% Triton X-100 for 1 h. Eventually, the sections were incubated in 1% Tween 20 for 10 min, and washed twice with PBS for 5 min. The parts were then stained with nucleus marker Hoechst, endothelial cell marker CD31 and adventitial fibroblast marker α-SMA to visualized specific components in the fluorescent images.

### 2.9. Statistical Analysis

Data are stated as mean ± standard deviation. Statistical calculation was showed using GraphPad Prism 5.0 (2007, GraphPad Software, San Diego, CA, USA) and the analysis of variance was used to analyze statistical significance and then took Tukey’s multiple comparison tests. Semi-quantitative data from the immunofluorescence staining and HE staining, which did not follow a normal distribution, were further analyzed through Mann–Whitney *U* test. Statistics for qPCR was performed using one-way ANOVA. Cell counts were measured by *t*-test. Unless otherwise noted, *p* < 0.05 was considered to make a difference statistically.

## 3. Results

### 3.1. Surface Morphology

**Figure 2:**
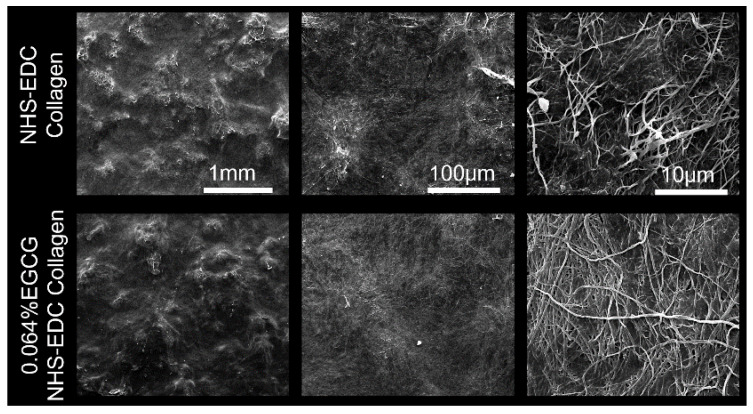
SEM images of EDC/NHS collagen membrane and 0.064% EGCG loaded EDC/NHS collagen membrane.

As shown in representative SEM images (Figure 2), the surface morphology of EDC/NHS collagen membranes exhibited a rough, disordered outlook, with fibers randomly distributed. After modified with 0.064% EGCG, there were no notable changes on the outlooks, while the arrangement of fibers appeared more ordered and intact. In addition, we also found that smaller fiber branches extend from the backbone when compared without EGCG treatment. These might be owing to the loading of EGCG hydrogen bonds formation between collagen fibers and EGCG molecules.

### 3.2. Mechanical and Chemical Properties

**Figure 3:**
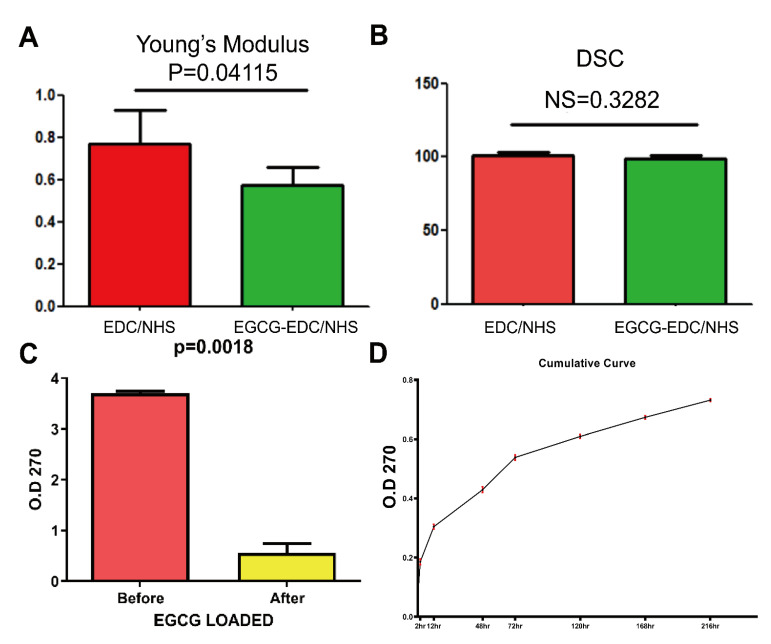
Young’s Modulus (**A**) and DSC statistics (**B**) of EDC/NHS collagen membrane and 0.064% EGCG-EDC/NHS collagen membrane. NS = no significance. (**C**) EGCG-EDC/NHS-Col fabrication assessment by detecting the OD value of EGCG solution before and after immersing membrane. (**D**) In vitro cumulative release profile of E-HA.

To examine the strength, stiffness, and elasticity of the membranes, measurement of US, UE, and YM were carried out and the corresponding volumes were written in Table 2. As US seemed relatively constant between the control and EGCG group, both UE and YM showed different trends. The modification of 0.064% EGCG strengthened the UE volume of collagen membranes, whereas weakened the YM volume (Figure 3A). Also, there was no statistical difference between membranes with or without EGCG modification in result of DSC measurement (Figure 3B). From the results of the FTIR spectra and DSC measurement, it could be concluded that chemical properties remain consistent before and after the treatment of 0.064% EGCG. The FTIR spectra indicated that no structural changes had occurred with collagen fibers since the absorption peaked at 1235 cm^−1^ (tertiary structure) and 1450 cm^−1^ (pyrrolidine ring vibrations) of EDC/NHS collagen membrane, and 0.064% EGCG-EDC/NHS collagen membrane remained constant, indicating that the secondary structure of collagen triple helix retained its integrity in both membranes and the great biological performance of collagen membranes. Also, the ratio of 1235 cm^−1^/1450 cm^−1^ (Table 3) came out to be around 1.00, illustrating that the secondary structure of collagen triple helix retained its integrity in both membranes, which assured the great biological performance of collagen membranes [21]. Therefore, it could be inferred that EGCG did not affect the physicochemical property of collagen.

The special properties of EGCG-EDC/NHS-Col may also be related to its release profile. After immersing EDC/NHS-Col into EGCG solution, EGCG-EDC/NHS-Col was fabricated successfully EGCG adhere to membrane, based on the OD value of the residual solution decreased, representing that the concentration reduced (Figure 3C). Since EGCG solutions had a maximum absorption wavelength of 270 nm at all concentrations when detected by ultraviolet-visible spectrophotometry, 270 nm was selected for detection. Cumulative release profile of EGCG-EDC/NHS-Col was obtained by detecting the amount of EGCG released to the buffer with time. As shown in Figure 3D, the slope of the cumulative curve demonstrated the release rate was non-constant. A great release amount could be seen in the first 2 h, and gradually decreased at 12 h, finally stabilized after 72 h. That is, after loading EGCG on the membrane, it can be released to the surrounding area over time. The release rate increases significantly in the initial stage and tends to be stable on the third day.

### 3.3. Cell Viability

**Figure 4:**
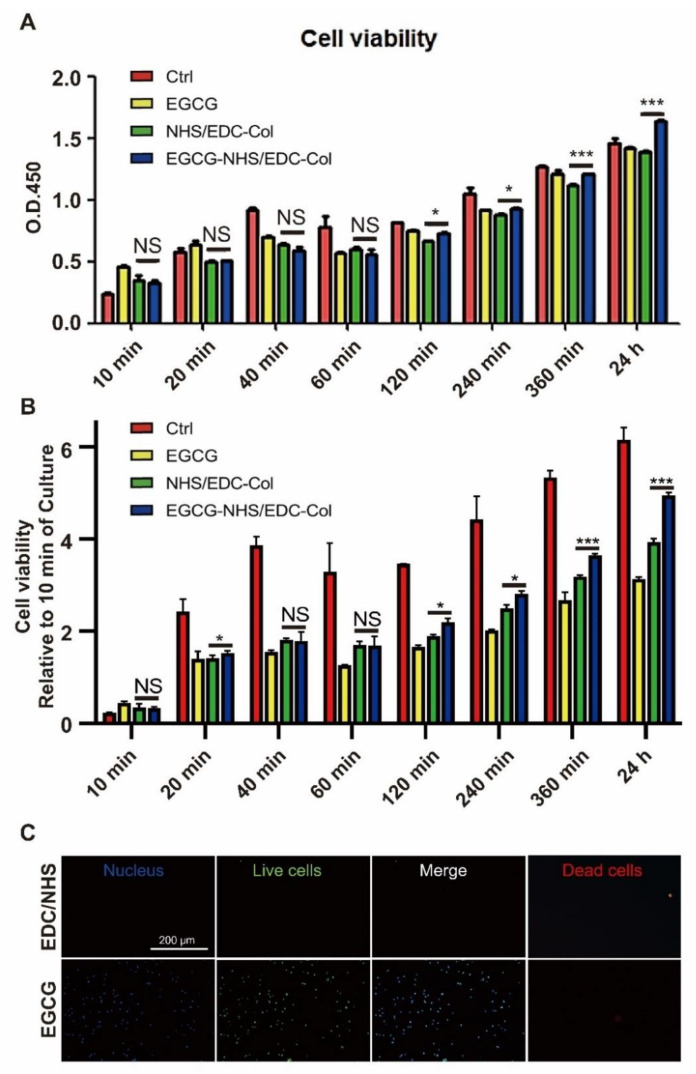
CCK-8 results (**A**), CCK-8 relative to 10 min results (**B**), and Calcein AM/Hoechst staining (**C**) of Raw 264.7 cultured on different conditions. Ctrl, standard medium; EGCG, 0.064% EGCG supplemented medium; EDC/NHS-Col, EDC/NHS collagen membrane; EGCG-EDC/NHS-Col, 0.064% EGCG-EDC/NHS collagen membrane. Green, live cells; blue, nucleus; red, dead cells. NS = no significance, * *p* < 0.05, *** *p* < 0.001, one-way ANOVA with Tukey’s multiple comparison testing, *n* = 3 in each group.

The results of CCK-8 staining showed the cell viability of Raw 264.7 cells under pure medium, 0.064% EGCG supplemented medium, EDC/NHS collagen membrane, and 0.064% EGCG-EDC/NHS collagen membrane (Figure 4A) from the first few minutes to 1 day. In the first hour, there was no significance between the groups. The improvement of cell viability by EGCG was raised after culturing for 2 h and the following time points. In order to further reflect the group differences at different time points, we compared and analyzed the OD value of the subsequent time period with the OD value of 10 min. It can be inferred that as culture time increases, relative OD value also increases significantly (Figure 4B). To verify the effect of EGCG on cell viability after culturing for a longer time, Calcein AM/Hoechst and PI staining was conducted on day 5 (Figure 4C). Statistical analysis was conducted on the number of cells in the five fields under the microscope to obtain the corresponding mean value and standard deviation. The *t*-test was further adopted. Ultimately, the number of dead cells in EGCG group was 2.4 ± 3.2, while EDC/NHS was 3.2 ± 2.2, accompanied with no statistical difference between the groups. The test results also showed the same trend with more live cells on EGCG-EDC/NHS-Col comparing with EDC/NHS-Col, indicating that the addition of EGCG was conducive to the performance of cell proliferating.

### 3.4. Cell Adhesion

**Figure 5:**
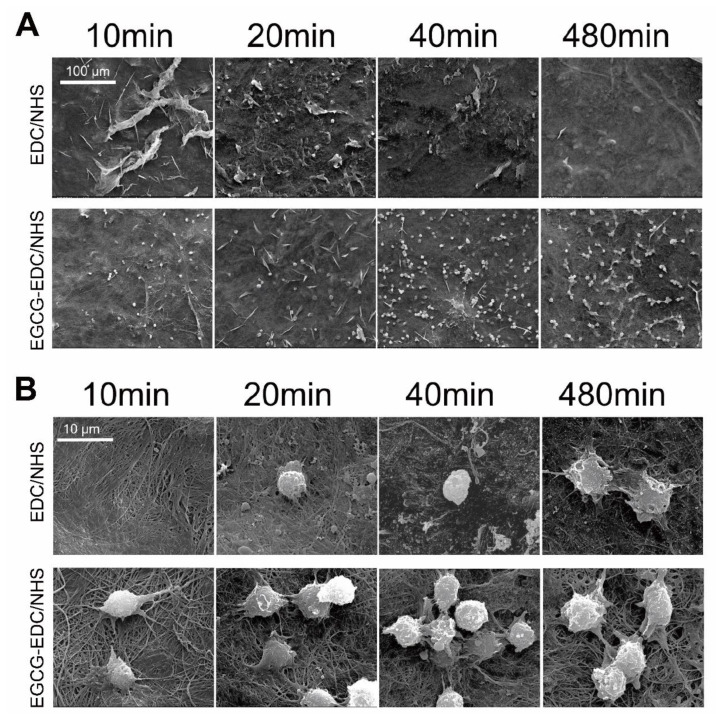

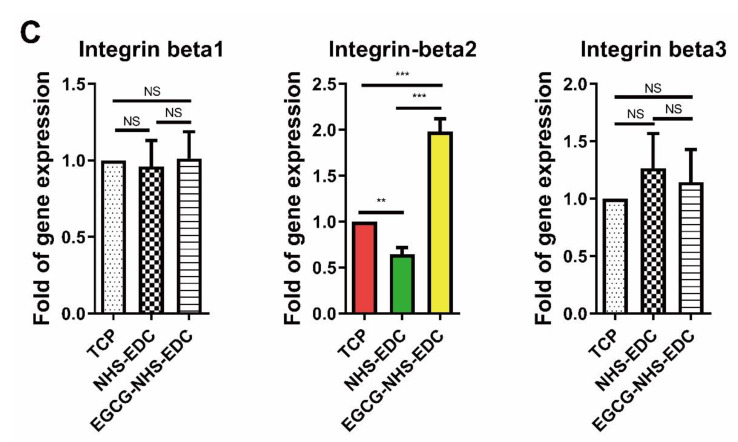
SEM images of Raw 264.7 cells cultured on EDC/NHS and EGCG treated EDC/NHS collagen membranes under low (**A**) and high (**B**) magnification, and the expression levels of integrin beta 1, 2, and 3, after culturing on TCP, EDC/NHS-Col and EGCG-EDC/NHS-Col for 10 min (**C**), respectively. NS = no significance, ** *p* < 0.01, *** *p* < 0.001, one-way ANOVA with Tukey’s multiple comparison testing.

In order to observe cell adhesion towards the membrane, SEM was used to observe the morphology of Raw 264.7 cells cultured on membranes in 8 h (Figure 5A,B). It elucidated membrane with the modification of EGCG, not only had more cells adhered to the surface but also a better spreading condition of cells was detected. Moreover, more protrusions could be seen to seize the fibers even at very early incubation, compared to that without EGCG. Considering integrin, the transmembrane molecules, with large extracellular domains that are reported to play a significant role in adhesion between cells and ECM [21,22], expression levels of integrin beta 1, 2, and 3 in Raw 264.7 cells were measured. The results showed there existed a boost of integrin beta 2 in the EGCG-EDC/NHS group, compared with group tissue culture plate (TCP) and NHS-EDC, whereas no differences appeared in the expression of integrin beta 1 and 3 with the treatment of 0.064% EGCG (Figure 5C). These results suggested that 0.064% EGCG could enhance the adhesion properties of EDC/NHS collagen membrane at early phases.

### 3.5. Promotion of Revascularization

**Figure 6:**
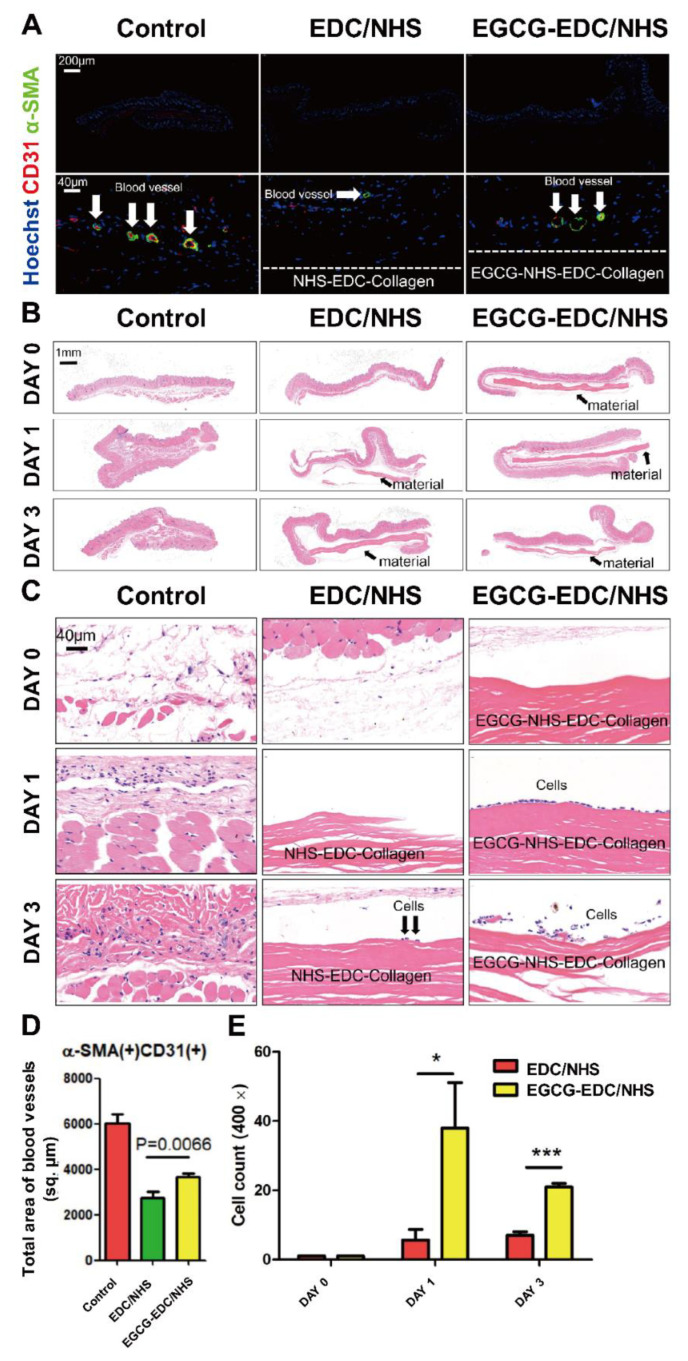
Results of subcutaneous implantation. Immunofluorescence staining of αSMA (green), CD31 (red), and Hoechst (blue) in subcutaneous implantation samples of EDC/NHS-Col and EGCG-EDC/NHS-Col, the control group refers to normal skin (**A**); semi-quantitative analysis of total blood vessel area was calculated in each group (**D**). Recruitment of monocyte/macrophage after subcutaneous implantation of EDC/NHS collagen membrane, EGCG-EDC/NHS collagen membrane and the control group underwent a sham procedure. HE staining of subcutaneous tissue (**B**,**C**) and cell counting of monocyte/macrophage (**E**) in NHS/EDC-Col and EGCG-EDC/NHS-Col were conducted on day 0, 1, and 3 post implantations. Arrows indicate material (**B**) and monocyte/macrophage (**C**). Statistical significance was analyzed through the analysis of variance followed by Tukey’s multiple comparison tests and Mann–Whitney *U* test (*n* = 5). Data are presented as mean + standard deviation. * *p* < 0.05, *** *p* < 0.001.

We identified the ability of 0.064% EGCG EDC/NHS-Col to promote revascularization through the immunofluorescence staining of nuclei, vascular endothelial cell marker CD31, and adventitial fibroblast marker α-SMA [23]. Preformed in Figure 6A, the growing number of the blood vessels were generated in EGCG-EDC/NHS-Col that compared to EDC/NHS-Col, meanwhile, as preformed in Figure 6D, the results of semi-quantitative analysis of total blood vessel area showed that it was larger in 0.064% EGCG EDC/NHS-Col than that in the EDC/NHS-Col (*p* < 0.01).

### 3.6. Recruitment of Monocyte/Macrophage

12 h after the implantation, there were no visible cells recruited at surgical sites, but with another 12 h, we could see that around the membrane, a certain number of immune cells were gathered, which was similar at three days post implantation (Figure 6C,D). In addition, semi-quantitative cell counting revealed that at 1- and 3-days post surgeries, 0.064% EGCG-EDC/NHS-Col recruited more monocytes/macrophages, while EDC/NHS-Col was about one-third the number of the experimental group (Figure 6E).

**Figure 7:**
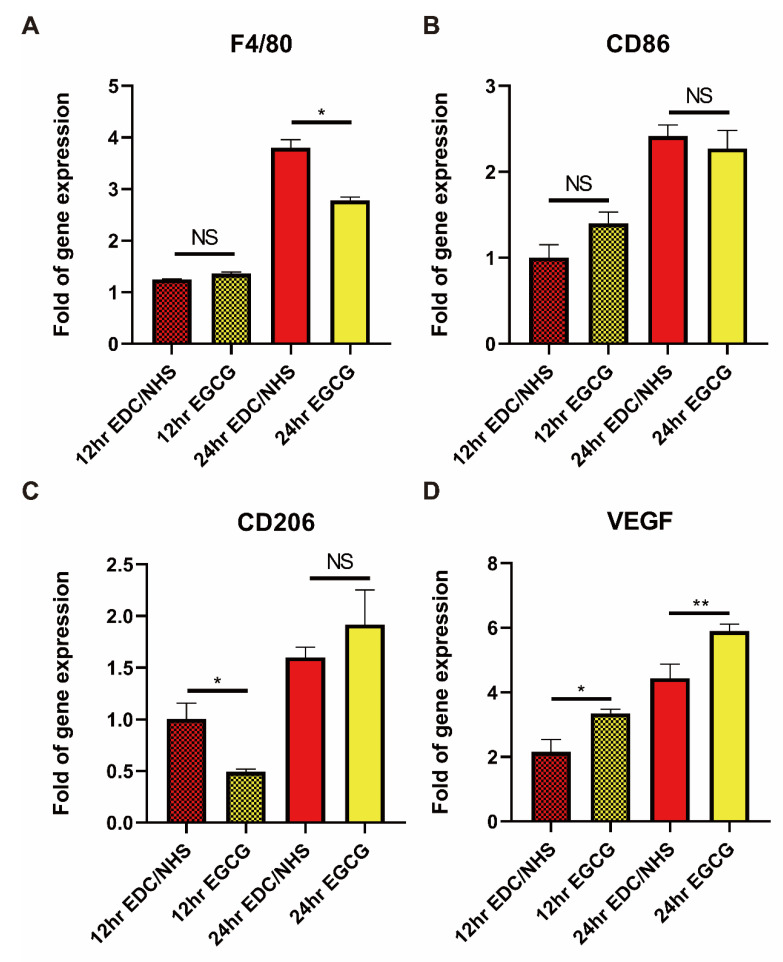
qPCR results of full-thickness skin defects in vivo at 12 and 24 h after implantation with EDC/NHS-Col and EGCG-EDC/NHS-Col. Marker genes included F4/80 (**A**), CD86 (**B**), CD206 (**C**), and VEGF (**D**). The macrophages were recruited and proliferate more in 24 h (**A**); the same trend could also be reflected in macrophage phenotypes (B&C); higher levels of angiogenesis-related expression markers VEGF showed in 24 h (**D**). NS = no significance, * *p* < 0.05, ** *p* < 0.01, one-way ANOVA with Tukey’s multiple comparison testing, *n* = 3 in each group.

Markers of macrophages and angiogenesis-related factor, VEGF, were detected at 12- and 24-h post membrane implantation below murine full-thickness skin defect (Figure 7). It was found that the detected markers between the two time points were statistically different with the increase of all targeted gene expression levels from 12 to 24 h, indicating that cells around the defect were quite active. Then, the effects of EGCG at the same time point were further explored, there was no difference in the expression of F4/80 at 12 h, while EGCG-EDC/NHS-Col expressed less F4/80 at 24 h, which may relate to the anti-inflammatory effect of EGCG (Figure 7A). However, the pro-inflammatory macrophage-related marker, CD86, showed no statistical difference at both time points, whereas pro-repair macrophage conventional marker, CD206, with a trend of gene expression transformed into not statistically significant, and the increase was relatively not obvious compared with other genes (Figure 7B,C). In addition, VEGF, as a very important upstream molecule for angiogenesis, was not only statistical different at both time points, but also with the largest increase, demonstrated the advantage of EGCG (Figure 7D).

## 4. Discussion

The oral cavity is one of the densest habitats of human bacteria and viruses, and it plays an active role in shaping the homeostasis of the oral cavity, especially in periodontitis [24]. The completion of the repair of oral soft and hard tissue defects in a complex and dynamic microenvironment depends on the capacity of implanted materials and the regulation of the local immune microenvironment including FBR. To reconstruct tissue deficiency, the application of membrane biomaterials and certain surgery like GBR and GTR are on account of its morphology. According to previous studies, the use of EDC as a crosslink agent could not only prolong the integrity of the membrane after implantation in different model, but also avoid severe FBR, short of vascularization in the early stage of poor integration and healing, which are the fatal flaws of traditional cross-linking agents [25]. The addition of NHS would improve EDC-mediated crosslinking, stabilize active intermediates, and reduce side products for subsequent reactions [26]. However, the present EDC/NHS crosslinked collagen membrane lacks the ability to modulate immune responses and induce immune-related cell behavior. Regarding cell behavior, EGCG, formation of hydrogen bonds between collagen and EGCG, has shown the ability to complement these two aspects in our previous research [20]. Thus, we attempted to modify to EDC/NHS crosslinked collagen membrane with 0.064% EGCG. After characterizing the material, results show that the framework of collagen remains integral and the strength of membranes is barely altered, whereas the stiffness is moderately enhanced, though elasticity is slightly weakened. These not only ensure the membrane will not be too supple to tightly barrier the soft and hard tissue, but also verify that the chemical structure related to the biological activity of collagen has not been destroyed [27].

Over the past decades, the ability of biomaterials to promote tissue regeneration by regulating immune cells in advance has been confirmed, the composition, physical and chemical properties, and surface morphology of which are the critical factors that affect FBR. As one of the dominant immune cells in FBR, macrophages can acutely polarize into different phenotypes according to the microenvironment created by the implant and direct the accumulated cells’ behaviors that strongly involve in tissue reconstruction [28]. The arrangement and diameter of the fibers can significantly affect the behavior of macrophages. Anisotropic membranes with thicker fibers have an increased tendency to oxidative degradation, compared with the thinner and isotropic one [29], more macrophages adherence on the align compared with the random and the smaller fibers also have been proved with better biocompatibility as thinner fibrous capsule and abundant volume of blood vessel formation [30]. In this article, we found that the surface morphology of EGCG-EDC/NHS-Col has been altered with smaller fiber branches extended from the backbone and the arrangement became more coherent, which may account for the cell viability and adherence. Under the electron microscope, the viability of RAW 264.7 on EGCG-EDC/NHS-Col is significantly higher than any other groups at all detection points during 2–24 h after implantation, the same results can be seen in CCK-8. Not only did more macrophages adhere to membrane with the treatment of EGCG, but the adhered macrophages were activated at an early stage (20 min) with many protrusions, and which is also confirmed by PCR detection. Also, more monocyte/macrophage recruitment could be seen on EGCG-EDC/NHS-Col in the subcutaneous implantation. As researchers have found that the onset of neovascularization greatly depends on macrophage and its coherent phenotypic switch [31,32], we highly believe the promising angiogenesis is the result from the recruitment of macrophages which cannot be excluded considering the microstructure of EGCG-EDC/NHS-Col. Of note, the modification of EGCG was highly competent at promoting vascularization that involved the secretion of M2-related chemokines [17]. The formation of blood vessel could offer nutrient, stem cells and oxygen supply, and waste discharge, vastly improving the regenerative response.

Therefore, our efforts are directed at macrophages and the outcome of vascularization as primary study towards the developed EGCG modified EDC/NHS collagen membrane. And our results showed that the modification of EGCG is beneficial to cell viability, adhesion, and vessel formation in both subcutaneous implantations in vivo and RAW 264.7 culture experiment in vitro. The great biocompatibility of EGCG modified EDC/NHS collagen membrane is stated without compromising the advantages of collagen itself. Furthermore, our results demonstrated the modified membranes could significantly affect the attachment of macrophages and enhance its viability both in vivo and in vitro, which could be related to the formation of vessels. Additionally, the results of qPCR showed that the macrophage related markers were significantly increased within 24 h. Although the phenotype could not be identified, the expression level of CD86 was higher than that of CD206 indicating both upregulating of M1 and M2 [33]. This could be related to the fact that the detection time points were all in the acute stage of injury (the macrophages accumulation in the early stage were mainly pro-inflammatory, while the macrophages were mainly pro-regenerative in the advanced stage). However, the angiogenic advantage of EGCG was also verified in qPCR, indicating that EGCG is promoted to repair and regeneration. Nevertheless, whether it is on account of the recruitment of specific phenotypes of macrophages and phenotypic transformation induced by EGCG needs further and long-time study. Moreover, the mechanisms of angiogenesis need further investigation and more evidence-based experiments. In short, we developed a biomaterial that can facilitate the early recruitment of macrophages and the formation of blood vessels that possesses great potential for tissue regeneration in the field of implant dentistry.

## 5. Conclusions

The modification of EGCG, EDC/NHS collagen, and GBR/GTR membrane can better promote macrophages’ adhesion and improve viability, which is confirmed in our experiments that the attachment of living cells and the expression of adhesion-related integrin is indeed increased. In addition, there was a statistically significant differences in angiogenesis and the marker gene of macrophages changing with EGCG loaded EDC/NHS cross-linked membrane implantation. Long-term foreign body immune response and the effect on bone regeneration still need more long-term research to confirm.

## Figures and Tables

**Table 1:** Nucleotide primers used for quantitative polymerase chain reaction.

Genes	Oligonucleotide Sequence
GAPDH	Forward: TGAAGCAGGCATCTGAGGG Reverse: TGAAGTCGCAGGAGACAACC
Integrin b1	Forward: CCAAGTGGGACACGGGTGA Reverse: CTGCTGCTGTGAGCTTGGTG
Integrin b2	Forward: GCAGCAGAAGGACGGAAACG Reverse: AGGGGGTTGTCGTTGTTCCA
Integrin b3	Forward: AGACAGCGCCCAGATCACTC Reverse: GCCAATCCGAAGGTTGCTGG
F4/80	Forward: ATTCCCGTGTTGTTGGTGG Reverse: TGCTTTGGCTGGATGTGC
CD86	Forward: TGTTTCCGTGGAGACGCAAG Reverse: TTGAGCCTTTGTAAATGGGCA
CD206	Forward: CTCTGTTCAGCTATTGGACGC Reverse: CGGAATTTCTGGGATTCAGCTTC
VEGF	Forward: ACACGGGAGACAATGGGATG Reverse: GGCAGGCAAAAGGACTTCG

**Table 2:** Mechanical properties of membranes.

Groups	Ultimate Stress (MPa)	Ultimate Elongation (%)	Young’s Modulus
EDC/NHS-Col	18.32 ± 3.12	22.44 ± 3.12	0.77 ± 0.16
EGCG-EDC/NHS-Col	18.18 ± 2.97	29.29 ± 2.78	0.57 ± 0.09

**Table 3:** FTIR ratio at the bands of 1235 and 1450 cm^−1^.

Groups	1235/1450
NHS/EDC-Col	1.001 ± 0.002
EGCG-NHS/EDC-Col	0.989 ± 0.003

## Data Availability

Data sharing is not applicable for this manuscript.
